# *Drosophila* FMRFa neuropeptide signaling modulates systemic glycogen metabolism and fitness in a sex-specific manner

**DOI:** 10.3389/fendo.2026.1764330

**Published:** 2026-05-12

**Authors:** Tetsuya Miyamoto, Veronika Mojik, Hubert Amrein

**Affiliations:** Department of Cell Biology and Genetics, Naresh K. Vashisht College of Medicine, Texas A&M University, Bryan, TX, United States

**Keywords:** Drosophila, fat body, flight muscle, FMRFa, glucose, glycogen, jump muscle

## Abstract

Glycogen is the major carbohydrate reserve in animals, mainly in the muscle and liver. It is mobilized via glycogenolysis to liberate glucose, which is used for glycolysis/mitochondrial respiration in the muscle to produce ATP, or released into the blood to maintain sugar homeostasis especially when nutrients are scarce. Although glycogen homeostasis is critical for animal physiology, the mechanisms controlling glycogen storage and utilization remain poorly understood. We recently showed that *Drosophila* FMRFa neuropeptide signaling is required for glycogen homeostasis in the jump muscle, a major tissue of *FMRFa receptor* (*FMRFaR*) expression. Here, we report that glycogen accumulation in the flight muscle and fat body also depends on FMRFa signaling, despite the absence of *FMRFaR* expression in these tissues. *In vivo* glucose imaging revealed that neither *FMRFa* nor *FMRFaR* mutants show deficits in intracellular glucose levels, consistent with a defect in glycogen synthesis or breakdown. Lastly, mutant flies exhibit reduced lifespan when kept on normal or protein-only food, but not on sugar-only food. These findings reveal that *Drosophila* FMRFa-FMRFaR signaling is a key modulator of systemic glycogen metabolism, thereby influencing survival and fitness in a diet-dependent manner.

## Introduction

Neuropeptides are highly conserved and present in virtually all animal taxa (1). In human, more than one hundred neuropeptides are expressed in diverse neuronal populations of both the central and peripheral nervous systems (2). Each neuropeptide acts through a specific receptor which may be expressed not only in neurons but also in peripheral tissues. Thus, neuropeptides can act as neurotransmitters to activate neighboring neurons (paracrine action), or they may function as neurohormones via the blood stream and activate cognate receptors in either distant neurons or other tissues (endocrine action), regulating many fundamental processes in metabolism and physiology (3).

Over 40 neuropeptide genes and a comparable number of cognate receptor genes have been identified in the insect model organism, *Drosophila melanogaster* (4). Many of these peptides are associated with behaviors such as feeding, aggression and mating, while other are critical for metabolic regulation including carbohydrate and lipid homeostasis. Some of the most extensively characterized neuropeptides involved in nutrient homeostasis are the *Drosophila* insulin-like peptides (Dilps) and Adipokinetic hormone (Akh). Dilps play critical roles in growth and stress response by regulating the metabolism of carbohydrates, proteins and lipids (5), whereas Akh promotes lipid mobilization, as *Akh* mutant flies display abnormal accumulation of triglyceride (6–8). Thus, Dilps and Akh have functions analogous to mammalian insulin and glucagon, respectively.

Recent studies have provided abundant evidence for tissue-to-tissue communication across animal species, involving not only signaling from the brain to peripheral tissues but also feedback from peripheral tissues to the brain, as well as crosstalk among peripheral tissues. Muscle has emerged as a critical hub for energy homeostasis in both vertebrates and invertebrates, with the identification of numerous myokines (9–12). In *Drosophila*, muscle has also been shown to secret signaling molecules that act on the brain, other muscle tissues, and the fat body to regulate growth, metabolism and immune response (13–17).

We previously identified a non-canonical role for Glucose-6-Phosphatase (G6P) in maintaining cellular glucose levels in specific subsets of peptidergic neurons in the central nervous system (CNS) (18). One of these subsets expresses FMRFamide (FMRFa) neuropeptides, and we showed that G6P promotes FMRFa secretion, which in turn is essential for maintaining glycogen homeostasis in the jump muscle (JM), where the FMRFa receptor (*FMRFaR*) is expressed (19). Moreover, restoring FMRFaR function specifically in the JM rescued the glycogen deficit of *FMRFaR* mutant.

In this study, we extended our analysis of FMRFa and its receptor beyond the JM. We show that FMRFa-FMRFaR signaling is also required for glycogen storage in the flight muscle (FM) and fat body (FB), even though neither of these tissues express *FMRFaR*. *In vivo* glucose imaging of *FMRFa* and *FMRFaR* mutant flies revealed that intracellular glucose levels were unchanged or elevated, suggesting that the deficit of glycogen is not due to lack in glucose supply. Interestingly, these mutants exhibit sex-specific differences in starvation resistance or survival when kept on protein-only or sugar-only diets. In addition, the mutants also showed reduced lifespan in both males and females kept on a complete diet, suggesting impaired overall fitness. Our findings uncover a novel regulatory mechanism by which FMRFa-FMRFaR signaling coordinates systemic glycogen metabolism and supports nutritional fitness in *Drosophila*.

## Results

**Figure 1:**
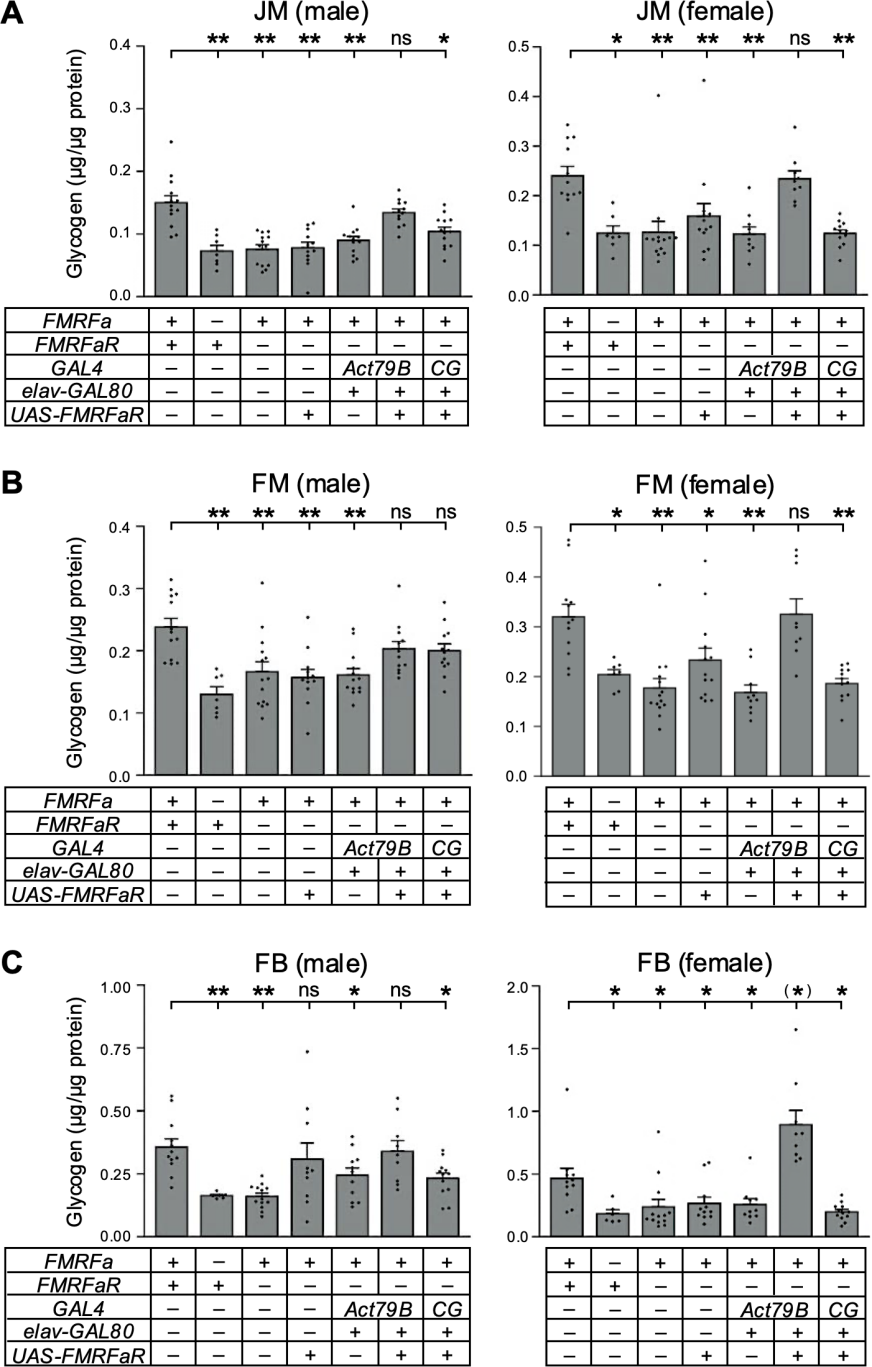
FMRFa signals through FMRFaR in the JM to establish glycogen stores in multiple tissues. Glycogen stores of *ΔFMRFa* and *FMRFaR^MB^* mutants were significantly lower compared to controls in the **(A)** JM, **(B)** FM, and **(C)** FB. In females, the defects of *FMRFaR^MB^* mutant were rescued across all tissues by expressing *UAS-FMRFaR* in the JM using *Act79B-GAL4* combined with *elav-GAL80* suppressor. In contrast, expression of *UAS-FMRFaR* in the FB using *CG-GAL4* did not restore glycogen levels in any tissues. Results in males were similar, except for some upregulations likely due to leaky *UAS-FMRFaR* expressions. Data for male JM are adapted from our previous work (19). Asterisks indicate *P<0.05, **P<0.001 by Kruskal-Wallis test with Dunn’s *post-hoc* test. n=6-14. Error bars represent standard error.

We previously showed that flies lacking *FMRFaR* exhibited significantly reduced glycogen stores in the JM, a major site of *FMRFaR* expression (19). We note that *FMRFaR* is also expressed in the CNS, but the expression is absent in other tissues known to store glycogen. Regardless, expression of *FMRFaR* in the JM of *FMRFaR* homozygous mutant flies restored glycogen stores in the tissue, whereas expression in the FB did not (19). Since FMRFa-FMRFaR signaling is necessary to maintain systemic glucose levels during starvation, we hypothesized that this pathway may also influence glycogen metabolism in other tissues, such as the FM, the most metabolically demanding tissue in the fruitfly, and the FB, the main energy storage organ that stores both lipids and substantial amounts of glycogen (10, 20). We quantified glycogen levels in the JM, FM and abdominal carcass (comprising mainly of FB) of control and homozygous mutant flies of *FMRFa* (*ΔFMRFa*) and *FMRFaR* (*FMRFaR^MB^*). Based on actin quantification of phalloidin-stained tissues, we estimated that abdominal wall muscle contributes less than 10% of the glycogen content in the abdominal carcass (19). Glycogen amounts were significantly lower in all three tissues of both mutants when compared to controls (**Figures 1A–C**). Notably, *GAL4*-mediated expression of *UAS-FMRFaR* in the JM (*Act79B-GAL4:elav-GAL80:FMRFaR^MB^ UAS-FMRFaR*) fully restored the glycogen storage deficit in *FMRFaR^MB^* mutants. Potential, low-level *GAL4* activity in the CNS was suppressed using *elav-GAL80*, confirming that restoration of glycogen levels in these flies was solely due to *FMRFaR* expression in the JM. These results indicate that FMRFa signaling systemically controls glycogen stores across metabolically highly active muscle (FM), as well as the main energy storage organ (FB), and that the *FMRFaR* expression in the JM alone is sufficient to maintain glycogen homeostasis in these non-*FMRFaR*-expressing tissues. Interestingly, restoring *FMRFaR* expression in the JM of *FMRFaR^MB^* mutant females resulted in complete rescue of glycogen content in the FB, whereas *CG-GAL4* driving *FMRFaR* expression in the FB failed to rescue the deficit (**Figure 1C**). These findings show that FMRFa-mediated regulation of systemic glycogen metabolism is coordinated through the JM, suggesting an unexpected role for this muscle in whole-body glycogen homeostasis.

**Figure 2:**
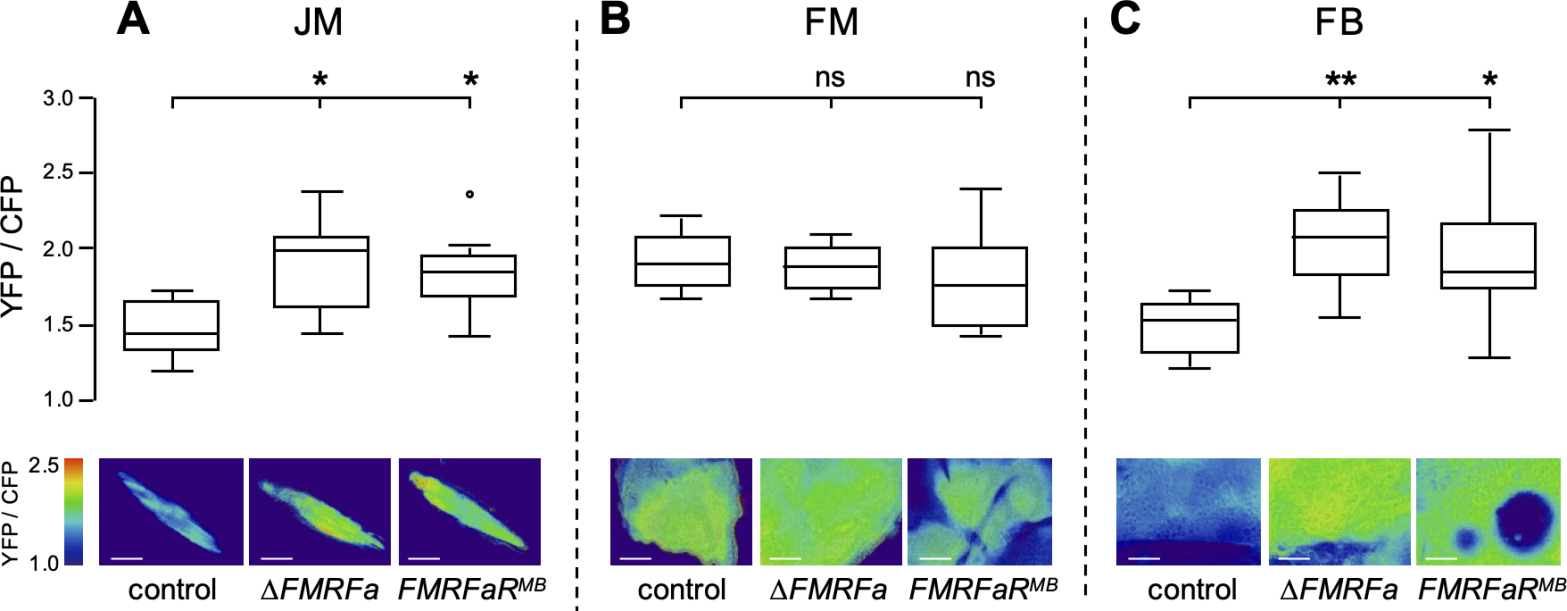
Δ*FMRFa* and *FMRFaR^MB^* mutants exhibit no defects in cellular glucose levels in all tissues. Intracellular glucose levels were measured using Glu700, a FRET-based glucose sensor, expressed in the JM and FM by *Act88F-GAL4* and in the FB by *CG-GAL4*. **(A)** In the JM (femur muscle), both *ΔFMRFa* and *FMRFaR^MB^* mutants showed higher glucose levels than controls. **(B)** In the FM (indirect flight muscle), glucose levels did not differ significantly among genotypes. **(C)** In the FB, both mutants showed higher intracellular glucose levels than controls. Scale bars represent 50 µm. Asterisks indicate *P<0.05 by Mann-Whitney U test. n=6-11. Error bars represent standard error.

Muscle and FB glycogen levels depend on availability of intracellular sugars, specifically glucose, as well as the activity of glycogen metabolizing enzymes. Thus, lower glycogen levels in FMRFa signaling defective flies could in principle be due to lack of sufficient glucose import into these tissues, reduced glycogen production (too low activity of Glycogen synthase, Glycogen branching enzyme) or accelerated glycogen breakdown (too high activity of Glycogen phosphorylase, Glycogen debranching enzyme). To assess whether glucose availability in these tissues contributes to their lower glycogen levels, or whether the glycogen deficits are solely due to misregulation of glycogen metabolism, we measured intracellular glucose levels *in vivo* using the FRET-based glucose sensor FLII^12^Pglu-700µδ6 (referred to as Glu700) (21). Intracellular glucose levels in the JM were significantly higher in the *ΔFMRFa* and *FMRFaR^MB^* mutants compared to those in the controls (**Figure 2A**). This finding rules out the possibility that reduced glycogen levels observed in *ΔFMRFa* and *FMRFaR^MB^* mutants results from insufficient glucose supply, instead, suggests a defect in glycogen synthesis or mobilization. It remains to be investigated whether elevated intracellular glucose levels are the result of increased glucose uptake or reduced glucose utilization. A similar trend was observed in the FB, where glucose levels were also elevated in both mutants (**Figure 2C**). However, glucose levels in the FM of mutants were comparable to those of controls (**Figure 2B**). We note that the control FM shows inherently high intracellular glucose levels (mean 1.9) compared to the JM (1.4) or FB (1.5), implying that the basal intracellular glucose level is upregulated, possibly due to the high energy demand of this tissue. Together, these results indicate that FMRFa signaling is dispensable for sugar supply but is essential for glycogen metabolism in multiple tissues.

**Figure 3:**
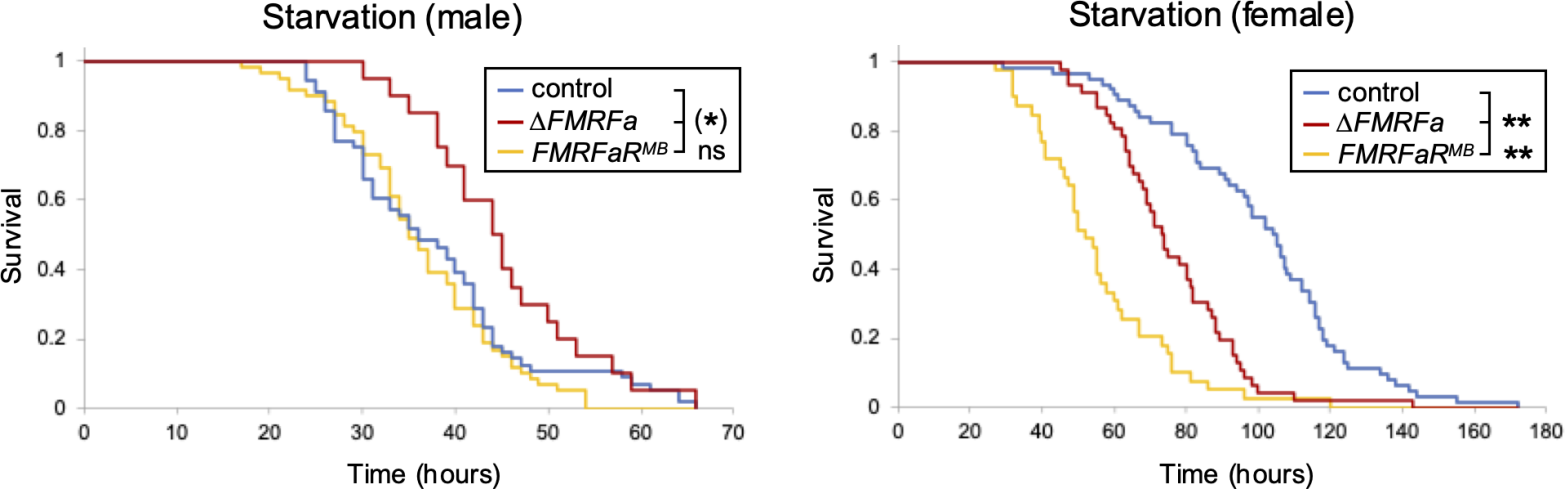
FMRFa signaling is essential for starvation resistance in females but not in males. Survival of controls, *ΔFMRFa* and *FMRFaR^M^*^B^ mutants was monitored under starvation conditions. In males, *ΔFMRFa* survived longer than control flies, whereas *FMRFaR^MB^* showed no significant difference. In females, both mutants exhibited significantly shorter survival times. Asterisks indicate *P<0.05, **P<0.001 using the log-rank test. n=17-45.

Glycogen homeostasis is vital for human health. A manifestation of impaired glycogen metabolism is glycogen storage diseases, which are characterized by hypoglycemia and muscle fatigue (22). In *Drosophila*, glycogen plays an essential role during development, as mutations in the genes encoding glycogen synthase and glycogen phosphorylase lead to larval lethality (23). Although *ΔFMRFa* and *FMRFaR^MB^* mutants are fully viable, we suspected that mutations in these regulatory genes may impair fitness and lifespan, especially when carbohydrates are restricted in the diet. To address this, we tested whether *ΔFMRFa* and *FMRFaR^MB^* mutants show reduced starvation resistance due to decreased glycogen stores. Homozygous *ΔFMRFa* and *FMRFaR^MB^* females showed significantly reduced starvation resistance compared to control females (**Figure 3**). In contrast, mutant males showed similar (*FMRFa^MB^*) or even increased (*ΔFMRFa*) starvation resistance when compared to control males (**Figure 3**). Although no sex-specific differences were observed in *FMRFa* or *FMRFaR* expression, these findings suggest that stored glycogen may be utilized in a sex-specific manner, whereby females relying more heavily on it than males.

**Figure 4:**
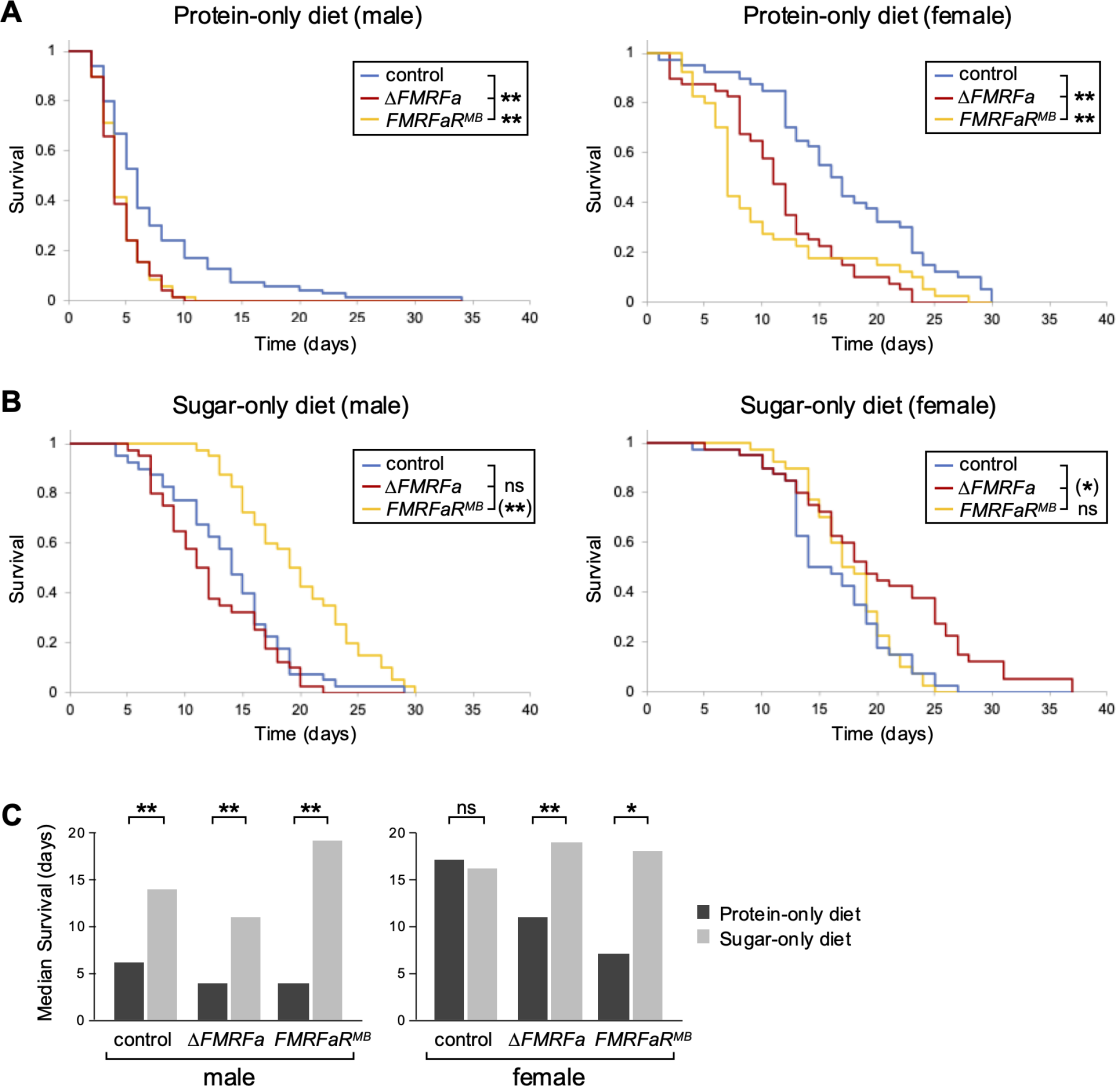
Diet and sex dependent effects of FMRFa signaling on survival. Survival of controls, *ΔFMRFa* and *FMRFaR^M^*^B^ mutants reared on protein-only or on sugar-only diets was monitored. **(A)** On the protein-only diet, both *ΔFMRFa* and *FMRFaR^M^*^B^ mutants showed reduced survival compared to control flies. **(B)** On the sugar-only diet, control flies did not show a survival advantage, and *FMRFaR^MB^* males (**P<0.001) and *ΔFMRFa* females (*P<0.05) survived significantly longer than control flies. **(C)** Comparisons of median survival times between diets in males and females. All flies except control females showed significantly longer survival on a sugar-only diet than on a protein-only diet. Asterisks indicate *P<0.05, **P<0.001 using the log-rank test. n=40-70.

Next, we investigated whether modulation of glycogen metabolism is essential for fitness under dietary stress conditions by monitoring survival of *ΔFMRFa* and *FMRFaR^MB^* mutant flies. When flies were maintained on a protein-only (casein-only) diet, median survival of control flies was extremely different between males (~ 6 days) and females (~17 days; **Figures 4A, C**), but both mutations reduced survival significantly in both sexes. When raised on a sugar-only diet (sucrose-only), neither males nor females deficient in FMRFa-FMRFaR signaling showed a significant reduction compared to controls (**Figure 4B**). In fact, mutant *FMRFaR* males and mutant *FMRFa* females had a somewhat longer survival than control males and females, respectively. These data indicate that the shortened survival of *ΔFMRFa* and *FMRFaR^MB^* mutants is diet-dependent and likely caused by impaired glycogen metabolism. Specifically, we posit that the sugar-only diet alleviates the systemic loss of glycogen stores by increasing circulating sugar levels, whereas the protein-only diet cannot compensate for that deficit. Of note, control females had a much longer median survival compared not only to *ΔFMRFa* or *FMRFaR^MB^* mutant females, but also control males reared on the protein-only diet (**Figure 4C**. Taken together, these results suggest that FMRFa signaling is especially critical for female to survive on sugar-deficient diets.

**Figure 5:**
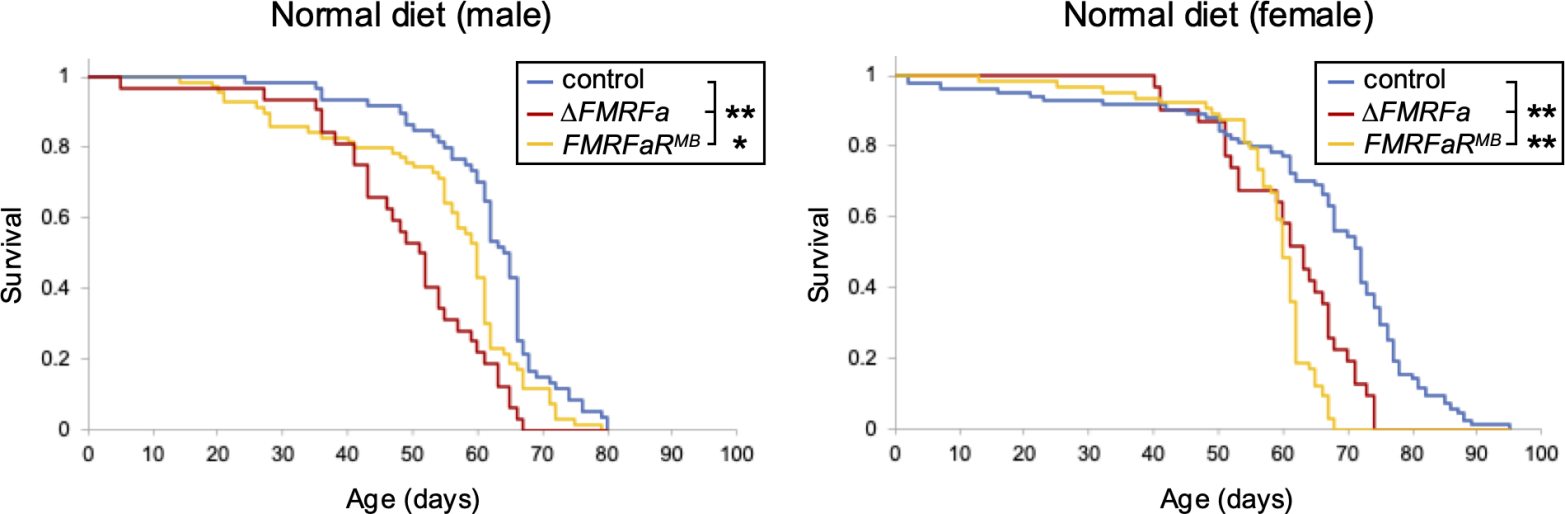
*ΔFMRFa* and *FMRFaR^MB^* mutants have reduced lifespans. *ΔFMRFa* and *FMRFaR^MB^* mutant flies showed significantly shorter lifespans than control flies reared on normal diet. Asterisks indicate *P<0.05, **P<0.001 using the log-rank test. n=31-84.

Lastly, we examined the effects of *FMRFa* signaling on lifespan when flies were provided with a complete diet (cornmeal, yeast and malt). Both *FMRFa* and *FMRFaR* mutants showed a reduced median lifespan by approximately 13 to 4 days in males and 9 to 12 days in females (**Figure 5**). While this reduced lifespan could be the result from the disturbed glycogen metabolism in mutants, it is also possible that the FMRFa signaling within the CNS contributes to this phenotype, since *FMRFaR* is abundantly expressed in neural tissue (19).

## Discussion

Glycogen serves as a key carbohydrate in most animal species, yet its homeostatic regulation remains poorly understood. We previously showed that *FMRFaR^2A-GAL4^* is expressed in the JM as well as in the CNS, and that FMRFaR is required for proper glycogen storage in the JM (19). Here, we extend this finding by demonstrating that *FMRFaR* expression in the JM is also required for maintaining glycogen levels in the FM and FB, the two other major organs known to store significant amounts of glycogen, but devoid of *FMRFaR* expression (**Figure 1**). Even ectopic expression of *FMRFaR* in the FB did not restore the glycogen deficit of *FMRFaR^MB^* mutants in this tissue. To our knowledge, JM-mediated signaling to maintain energy metabolism is a precedent and suggest that this tissue occupies a central role in balancing energy needs at the whole animal level.

What might be the mechanism by which the JM serves as a hub for whole animal glycogen homeostasis? One possibility is that activation of FMRFaR leads to the secretion of other signaling factor(s), such as a myokine, that acts on cognate receptors in the FM and FB. More than 650 myokines have been identified in humans, where they regulate diverse aspects of metabolism and energy homeostasis (11). In *Drosophila*, several muscle derived factors have been shown to regulate lipid metabolism in the fat body (13–15). Although the close anatomical proximity of JM and FM enables direct intercellular communication, including via gap junctions, communication between JM and FB must rely on humoral factors. Future studies will be necessary to identify candidate factors that convey glycogen homeostasis from the JM to other tissues in the fruit fly.

*In vivo* glucose imaging using a FRET-based fluorescent sensor, previously developed for neuropeptide neurons, enabled us to quantify intracellular glucose levels in non-neuronal tissues, including muscle and fat body. Both *ΔFMRFa* and *FMRFaR^MB^* mutants showed comparable or elevated glucose levels, indicating that the reduced glycogen content in these tissues is not caused by lack of glucose, but the result of either reduced production or accelerated breakdown of glycogen (**Figure 2**). This could arise from altered activity of glycogen synthase or glycogen phosphorylase, whose activities in mammals are known to be regulated by post-translational modifications (22). Identifying the downstream targets of FMRFa signaling in glycogen metabolic enzymes will be an important step toward understanding this regulation.

We note that in the control flies, the intracellular glucose level was higher in the FM than in the JM or FB (**Figure 2**). The FM consumes more energy to sustain flight than any other tissue, and therefore elevated glucose levels may reflect the need for elevated glycolysis and the generation of large amounts of pyruvate, a key substrate in mitochondrial oxidative phosphorylation. This observation aligns with previous studies showing that the indirect flight muscle has an exceptionally high energy consumption rate (24). Regardless, the elevated glucose levels in the FM is markedly different from the case in mammals, where the intracellular glucose concentration in the skeletal muscle is much lower than in the liver (25). Given that both muscle types have high energy needs, it is curious how different species accomplish this task through such contrasting intracellular glucose levels.

The broad deficit of glycogen stores in *ΔFMRFa* and *FMRFaR^MB^* mutant flies prompted us to examine the fitness of these mutants under various dietary conditions. Our analysis has shown that survival on protein-only diet was significantly reduced in both *ΔFMRFa* and *FMRFaR^MB^* mutants (**Figure 4A**). In contrast, survival on sugar-only diet was not impaired in mutants when compared to control flies with intact FMRFa-FMRFaR signaling (**Figure 4B**). These results indicate that FMRFa signaling plays an important role in *Drosophila* fitness via regulating systemic glycogen metabolism in a diet-specific manner. We speculate that the sugar-only diet rescues the systemic loss of glycogen stores by increasing circulating sugar levels, whereas the protein-only diet cannot compensate for that deficit. This mirrors the treatment of hypoglycemia in Glycogen Storage Disease I patients through frequent carbohydrate feeding (22).

We expected lower glycogen levels in *ΔFMRFa* and *FMRFaR^MB^* mutants to reduce starvation resistance, given that flies mobilize substantial amounts of glycogen under nutrient deprivation conditions (26). Surprisingly, there was a clear sex-specific component in starvation resistance, with only females deficient in FMRFa-FMRFaR signaling showing reduced survival (**Figure 3**), even though glycogen levels were significantly reduced in both sexes (**Figure 1**). It is possible that male flies rely more on triglyceride for starvation, as suggested by rapid upregulation of lipase genes transcription when subjected to starvation (27).

Sex-specific differences were also observed in control flies maintained on restricted diets. Notably, control males died much earlier on the protein-only diet than on the sugar-only diet while control females showed no significant difference in survival (**Figure 4C**). This tendency was less pronounced in either *ΔFMRFa* or *FMRFaR^MB^* mutant flies, suggesting that FMRFa signaling contributes to sex-specific regulations of nutrient metabolism. *Drosophila* exhibit sexual dimorphism in feeding behavior and metabolism in response to protein ingestion, likely reflecting different reproductive demands. This is mediated by the endocrine system includes fat body-derived female-specific independent of transformer (FIT) and enteroendocrine cell-derived CCHamide1 (28–30). It will be interesting to determine whether FMRFa exerts sexually dimorphic effects by itself or interacts with other signaling systems.

Lifespan experiments demonstrate that FMRFa signaling contributes to fitness (**Figure 5**). Since glycogen levels depend on a dynamic balance between synthesis and breakdown, disruption of this equilibrium might cause fluctuations in circulating sugar levels, increase metabolic stress, and consequently result in a shorter lifespan. However, it is important to note that the *FMRFaR* is also abundantly expressed in the CNS, where it might modulate other physiological processes (19). Indeed, FMRFa signaling has been associated with other processes, including flight, sleep, escape response and cellular glucose uptake (31–34). Interestingly, brain FMRFa signaling has been reported to suppress protein intake through modulation of dopamine neurons in male flies (35). This raises the possibility that reduced lifespan in FMRFa signaling deficient flies could be the result of protein overconsumption (36). Although we did not observe any feeding defects in *ΔFMRFa* or *FMRFaR^MB^* mutants, subtle differences could have a significant impact on lifespan. Reliable tissue-specific manipulation of *FMRFaR* will be important for distinguishing its neural and peripheral functions in future studies.

## Materials and methods

### Fly strains and maintenance

Experiments were performed using adult *Drosophila melanogaster*, aged between 7–14 days. Flies were reared on standard fly food containing cornmeal, yeast and malt at 23-24˚C. The *yellow* and *white* (*yw*) was used as control flies, and both *FMRFa* and *FMRFaR* mutants were backcrossed five times to the *yw* strain, in order to generate comparable genetic backgrounds. The *yw; FMRFa^175^* mutant, referred to as Δ*FMRFa*, was generated previously in our laboratory (19). The *yw; FMRFaR^MB04659^* line, referred to as *FMRFaR^MB^*, was obtained from the Bloomington Drosophila stock center (stock #24212).

### Quantification and statistical analysis

Statistical significance for pairwise comparisons was determined using the Mann-Whitney U test, and for multiple comparisons using the Kruskal-Wallis test followed by Dunn’s *post-hoc* test. Survival and longevity analyses were performed using Kaplan-Meier method, and significance was assessed using the two-sample log-rank test. Statistical significance levels were indicated as *p<0.05, **p<0.001, and ns (not significant). All replicates represent independent biological samples (different flies or tissues). Statistical analyses were conducted using the Real Statistics add-in for Microsoft Excel.

### *In vivo* glucose imaging

Glucose imaging of the JM, FM and FB was performed as previously described for single neurons, with tissue-specific modifications. For the JM, the foreleg was cut at the coxa and mounted with a small drop of liquid silicon (Dow Corning, Sylgard 184 Silicone Elastomer base) on a glass-bottom dish (MatTek corporation, #P35G-0-10-C). The femoral muscle was imaged without further dissection. For the FM, the dorsal thoracic cuticle was removed to expose the indirect flight muscle, and the fly was positioned upside down on the dish with liquid silicone. For the FB, the dorsal side of abdomen carcass (with gut and reproductive tissues removed) was placed on the dish with liquid silicone. All dissection was performed dry, without any liquid medium and dissected tissue was immediately placed in liquid silicone to preserve intracellular physiological integrity.

Imaging was performed using a Nikon Eclipse Ti inverted microscope with a 20x water objective, a dichroic filter (Nikon; 89002), excitation and emission filter wheels with four Chroma filters ET430/24x (#234435), ET500/20x (#235394), ET470/24m (#234331) and ET535/30m (Chroma #239226). The light source was a Lumen 200 lamp (Prior Scientific Inc). Images were acquired using NIS-Elements software (Nikon). For each data point, three sequential images were captured to calculate FRET signals: 420–445 nm for CFP excitation and 458–482 nm for CFP emission with 400 ms exposure (Dd), 420–445 nm for CFP excitation and 520–550 nm for YFP emission with 100 ms exposure (Da), 491–508 nm for YFP excitation and 520–550 nm for YFP emission with 100 ms exposure (Aa). Dd and Aa values were used to correct false FRET signals caused by CFP and YFP alone. Spillover factors for CFP (0.290) and YFP (0.095) were experimentally determined (18). FRET efficiency was calculated using the following formula; (Da-0.29xDd-0.095xAa)/Dd. Image analysis was performed using the NIS-Elements software suite.

### Glycogen measurement

Adult flies were anesthetized with CO_2_. The abdomen (after removal of gut and reproductive organs) and the thorax (separated into dorsal and ventral halves corresponding to the FM and JM, respectively) were placed in 1.5 mL microtubes containing 50 µl of 0.2% NP-40. Samples were homogenized with a plastic pestle, and debris was removed by centrifugation at 14000 rpm for 20 minutes at 4°C.

For glycogen quantification, 5 µl of the supernatant was incubated with 5 µg of amyloglucosidase (Sigma-Aldrich, #A1602) with 50 mM sodium acetate buffer for 30 minutes at 37°C. The reaction mixture was then mixed with 100 µl of Infinity™ Glucose Hexokinase Reagent (Thermo Scientific, #TR15421) and incubated for 10 minutes at 37°C. Absorbance was measured at 340 nm. Glycogen amount was obtained by subtraction of free glucose which was determined by measuring 340 nm absorbance of 5 µl of the same sample without amyloglucosidase.

Total protein amount in each sample was determined using 1.25 µl of the same supernatant mixed with 100 µl of Bradford reagent (Bio-Rad, #5000205), and absorbance at 595 nm was compared to a standard curve. Glycogen values were normalized to the total protein amount per sample.

### Actin quantification in each tissue

The relative muscle content in the jump muscle, flight muscle and fat body was estimated based on actin levels visualized by phalloidin staining. Whole flies were fixed in 4% paraformaldehyde with 0.2% Triton X-100 for 5 days at 4°C. The fixed samples were cut along the mid-sagittal plane using a surgical blade. Hemisections were stained with 20 µM Alexa Fluor 555-conjugated phalloidin (Invitrogen A34055) for 40 minutes at room temperature, washed three times with PBS, and imaged using confocal microscopy. The Alexa Fluor 555 signal intensity was quantified with ImageJ.

### Starvation assay

Approximately 10 adult males or females were placed in separate petri dishes (55/15 mm) containing 1% agarose. Flies were monitored every hour using a CMOS camera (ELP, #USBFHD06H-SFV) controlled by Yawcam software (http://www.yawcam.com), and the number of live and dead flies were counted at each time point. Data was presented by Kaplan-Meier survival curves.

### Life span and survival assay

For lifespan assays, approximately 10 adult males or virgin females aged 2–5 days were housed in standard vials containing standard laboratory diet. Flies were kept at 25°C on 12 hours day-night cycle and transferred weekly (lifespan) or every 3 to 5 days (sugar-only and protein-only diets). Survival was monitored daily, and dead flies were recorded.

### Diets

The standard laboratory diet is comprised of 5.2% cornmeal (Genesee #66-101), 2.75% yeast (Genesee #62-108), 11% malt (Alternative Beverages #DME-L55) and 0.725% agar (Genesee #66-102) with 0.14% Tegosept (Spectrum #BE159). The protein-only diet contained 50 mg/mL casein (Alfa Aesar, #A13707), and the sugar-only diet contained 100 mM sucrose (Macron, #8360-06) in 1% agarose.

## Data Availability

The raw data supporting the conclusions of this article will be made available by the authors, without undue reservation.
