# Correction: *Drosophila* FMRFa neuropeptide signaling modulates systemic glycogen metabolism and fitness in a sex-specific manner

**DOI:** 10.3389/fendo.2026.1879789

**Published:** 2026-05-28

**Authors:** Tetsuya Miyamoto, Veronika Mojik, Hubert Amrein

**Affiliations:** Department of Cell Biology and Genetics, Naresh K. Vashisht College of Medicine, Texas A&M University, Bryan, TX, United States

**Keywords:** Drosophila, fat body, flight muscle, FMRFa, glucose, glycogen, jump muscle

The title of this article was erroneously given as: “*Drosophila* FMRFa neuropeptide signaling modulates systemic glycogen metabolism and fitness in a diet-dependent manner”. The correct title of the article is “*Drosophila* FMRFa neuropeptide signaling modulates systemic glycogen metabolism and fitness in a sex-specific manner”.

The original version of this article has been updated.

